# Undervalued Spiny Monkey Orange (*Strychnos spinosa* Lam.): An Indigenous Fruit for Sustainable Food-Nutrition and Economic Prosperity

**DOI:** 10.3390/plants10122785

**Published:** 2021-12-16

**Authors:** Abiodun Olusola Omotayo, Adeyemi Oladapo Aremu

**Affiliations:** 1 Food Security and Safety Niche Area, Faculty of Natural and Agricultural Sciences, North-West University, Private Bag X2046, Mmabatho 2745, South Africa; 2 Indigenous Knowledge Systems Centre, Faculty of Natural and Agricultural Sciences, North-West University, Private Bag X2046, Mmabatho 2745, South Africa

**Keywords:** fruit tree, food policies, food security, Loganiaceae, nutrients, market economies, novel products

## Abstract

*Strychnos spinosa* Lam. is among the top nutrient-dense indigenous fruit species that are predominant in Southern Africa. It is a highly ranked indigenous fruit based on the nutrition and sensorial properties, which make it an important food source for the marginalized rural people. On the basis of the high vitamin C, iron, and zinc content, it has the capacity to improve the food- nutrition and the socioeconomic status of individuals, especially those in the rural areas of the developing nations. The nutritional composition of *Strychnos spinosa* compare favorably with many of the popular fruits, such as strawberries and orange. Additionally, *Strychnos spinosa* has antioxidant activity similar to well-known antioxidant fruits, which keeps it in the class of the popular fruits, giving it added nutrition–health-promoting benefits. In order to improve the availability of *Strychnos spinosa*, more research on the domestication, processing, preservation, value chain, and economic potential need to be further explored. Therefore, we recommend more concerted efforts from relevant stakeholders with interest in *Strychnos spinosa* fruit production as a possible sustainable solution to food shortage, food-nutrition insecurity, malnutrition, and austerity, mainly in the rural communities of the developing countries.

## 1. Introduction

With the declined rate of global undernourishment (15% within 2000–2004 and 8.9% in the year 2019), about 690 million individuals remain undernourished globally. Meanwhile, the stunting rate further fell from 33% of children under age five in 2000 to 21.3% in 2019 [1,2,3]. In order to achieve the goal of ending undernutrition by the year 2030, there is need to encourage the consumption of a balanced diet, especially in the rural communities of the developing nations [4,5,6,7]. Interestingly, existing literature have ascertained that indigenous fruits are used to cover food lack and shortages, thereby, these remain a key option for dealing with micronutrient shortages during vulnerable times [8,9]. Indigenous fruits have been utilized in several ways since time immemorial for food needs of the local societies [3].

Generally, the potential of many indigenous fruits is underexplored, especially in the area of their basic botany, horticulture, food science, and economic value [10,11,12,13]. Indigenous fruits have the potential to provide the necessary phytonutrients required in the diet for food-nutrition security and the income of rural communities where the cultivation of the popular fruit species is not common [14]. In the warmer temperate regions of the globe, an indigenous fruit tree that stands out with a rich source of phytonutrients is the *Strychnos spinosa* [15]. It is one of the most important edible indigenous fruit trees in the wild. The fruit-bearing species of *Strychnos* belong to the family Loganiaceae. The tree has the capacity to stay edible in tropical heat, which is an important characteristic for food and nutrition security, as this will enhance availability and productivity [16,17,18].

In traditional medicine, *Strychnos spinosa* is often used in the treatment of venereal diseases, stomach-related aches, and snake bite attack [19]. *Strychnos spinosa* is known as a native or introduced species in many African nations. The plant has been reported across different African regions, including Southern Africa, East Africa, and West Africa [20]. In South Africa, *Strychnos spinosa* grows well in four provinces (Eastern Cape, Limpopo, KwaZulu-Natal, and Mpumalanga).

Furthermore, the conservation status of *Strychnos spinosa* is categorized as “least concern”, as its distribution and abundance possess a low risk of extinction [21,22]. However, the plant has a recent record of declining occurrence in Benin and Burkina Faso (West Africa), which was attributed to factors such as agricultural activities, urbanization, and animal breeding, rather than climate change and its impact [23]. Although the distribution and availability of the *Strychnos spinosa* is uneven in Africa, its food-nutritional and economic potentials suggest the need for a more conscious and holistic conservation approach.

*Strychnos spinosa* has several local uses, and it is known to be a rich source of nutrition and phytochemicals, thereby suggesting its potential health benefits [21,24,25]. Given the increasing importance of *Strychnos spinosa* in food-nutritional sovereignty, as well as its ecological advantage [26,27,28], this review provides an appraisal on the potential for sustainable food–nutrition and economic prosperity of *Strychnos spinosa*. It is anticipated that consolidated information on *Strychnos spinosa* is important in an attempt to unfold its nutritional and economic potential.

## 2. Method for Literature Search

The approach described by Omotayo et al. [29] was employed in literature selection. Different online sources, theses, dissertations, and research reports were explored. We searched online sources such as Web of Science (WOS), Google Scholar, PubMeb, and Scopus, using various terms and phrases. Examples of these include “*Strychnos spinosa*”, “Monkey orange”, “nutritional value composition *Strychnos spinosa*”, “ethno-medicinal importance of the *Strychnos spinosa*”, “uses of *Strychnos spinosa*”, and “description of *Strychnos spinosa*”. For this review, the focus of the search was on Africa, southern Africa, and South Africa from the year 1962 to December 2021.

**Figure 1:**
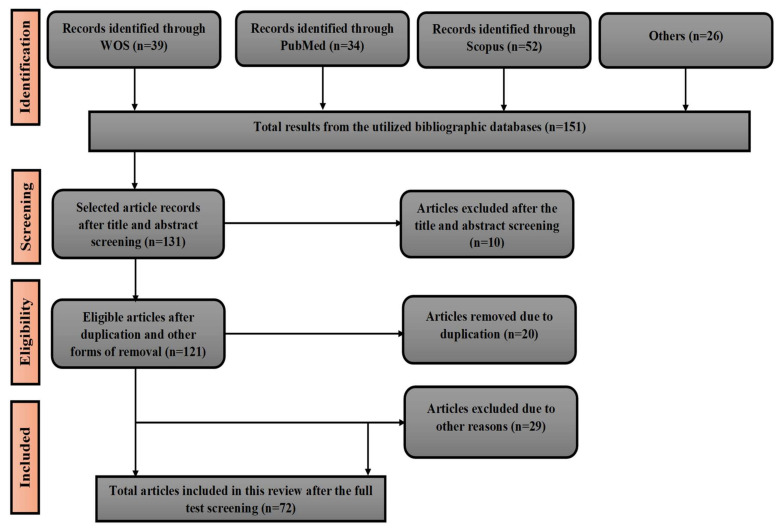
Preferred reporting items for systematic reviews and meta-analyses (PRISMA) for the exclusion and inclusion of articles.

For the search, studies that fit the inclusion criteria were derived in order to explore the content. The five areas explored and categorized were (i) distribution and description of *Strychnos spinosa*, (ii) uses of *Strychnos spinosa*, nutritional and phytochemical content (iii) economic potential (iv) postharvest handling, preservation, storage and processing, and (v) domestication of *Strychnos spinosa*, cultivation problems, and future research direction (Table 1). In this review, a sum of 151 peer-reviewed papers were retrieved that focused on *Strychnos spinosa*. Finally, an estimated 47.68% (72) of the literature was relevant, utilized, and included in the review article (Figure 1).

## 3. Botanical Description and Taxonomy of *Strychnos spinosa*

**Figure 2:**
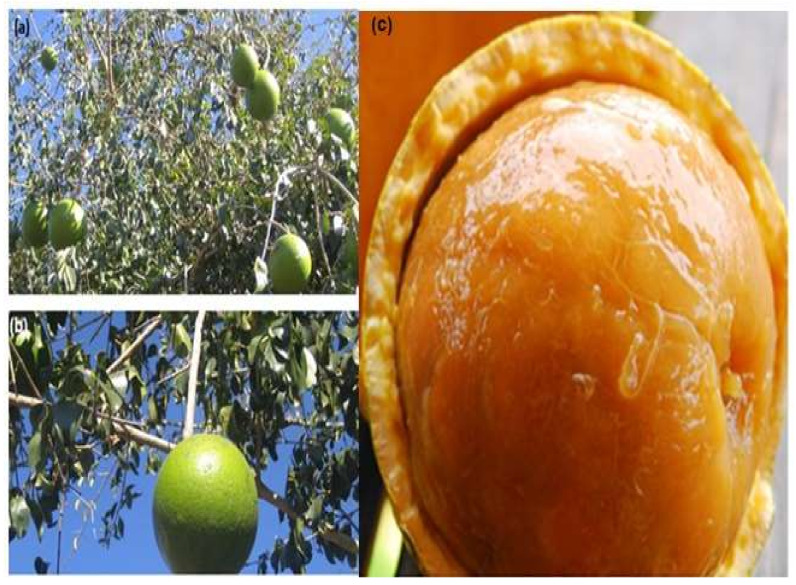
Morphology of *Strychnos spinosa.* (**a**) tree at fruiting stage; (**b**) mature green fruit; (**c**) ripe fruit.

About 75 species of *Strychnos* exist in Africa, with 20 species (e.g., *Strychnos innocua, Strychnos cocculoides, Strychnos pungens*, and *Strychnos spinosa*) producing consumable fruits in drought-prone and semi-arid areas [18,30,31]. *Strychnos spinosa* is a small tree of 1–7 m height, having straight and curved axillary spines, as well as a corky back [32]. The leaves are simple and oval (Figure 2a,b). The fruit is edible, round-shaped, 6–15 cm in diameter, and resembles a typical orange [31,33]. The unripe fruits (Figure 2b) are green, with wood peel of 34 mm that becomes yellow (Figure 2c) when ripe [31].

*Strychnos spinosa* fruit has a juicy, sweet-sour pulp, which is pale brown, with about a 3 cm flat seed, slightly similar to apricots [34]. *Strychnos spinosa* grows in well-drained soils [33,35]. Fruit weighs between 145 and 383 g, while about 300–700 fruits (40–100 kg) can be produced per tree stand. *Strychnos spinosa* is a seasonal fruit tree that is harvested between August and December [31]. However, the domestication of *Strychnos spinosa* remains in experimental stages, which is still a problem associated with its commercial prospect. Presently, *Strychnos spinosa* can be propagated via seeds, grafting, or budding, with the production of fruit starting 3–5 years after planting [27].

## 4. Nutritional and Phytochemical Content of *Strychnos spinosa*

### 4.1. Nutritional Composition of the Strychnos spinosa

*Strychnos spinosa* fruit contain energy, fibers, crude protein, and minerals (Table 2) [18]. Compared to other fruits, the vitamin C content for *Strychnos spinosa* is similar to that of oranges (*Citrus sinensis*) (50 mg/100 g) and strawberries (*Fragaria ananassa*) (59 mg/100 g) [18,31]. Therefore, the consumption of *Strychnos spinosa* provides a source of ascorbate and may alleviate nutrition insecurity for local communities. Most importantly, its fruit pulp (Figure 2c) can be sun-dried as a food preserve, thereby extending shelf-life and availability.

*Strychnos spinosa* fruit is a good dietary source of carbohydrates and proteins. Furthermore, it contains important minerals, namely iron, zinc, copper, and manganese [37], thereby suggesting that the consumption of *Strychnos spinosa* may serve as a source to meet the body requirement of zinc, iron, copper, and manganese. The deficiency of micro-minerals in the human body impairs growth and increases the susceptibility of such individuals to infections and risk of mortality, especially in children [38]. Although the presence of these aforementioned minerals in *Strychnos spinosa* fruit has been indicated, a wide variability in concentrations for some of them as reported by Lockett, et al. [39].

### 4.2. Phytochemicals in Strychnos spinosa

Phytochemicals are biological active compounds, such as the flavonoids and phenolic acids, with health-promoting values, such as anti-ageing and inflammation [18,21,40,41], which were mainly attributed to their ability to scavenge free radicals [18,42,43]. The rich phytochemicals that are abound in different parts of *Strychnos spinosa* remain key to explaining their food-nutritional benefits and future potential [44,45,46,47]. Diverse phytochemicals were confirmed in the leaves, branches, seeds, and fruit pericarp of *Strychnos spinosa* (Table 3). In addition, significant amount of phenolics and flavonoids were detected in the root-bark [37,48]. 

### 4.3. Physicochemical Properties of Strychnos spinosa

*Strychnos spinosa* fruit shows a delicate complex of aroma volatiles that are identified as a mixture of apricot, clove, pineapple, and citrus [26,33]. The degree of *Strychnos spinosa* ripeness influences the taste and sugar profile that varies based on the environmental-related factors [18]. Based on existing studies (Table 4), a wide variation have been confirmed in *Strychnos spinosa* [18,31]. The presence of organic acids in *Strychnos spinosa* is explained by the acidic content that blends with sugars, thereby making the plant to exert a blended acid-sweet taste [18]. The partial solubilization of the pectin and cellulose by the plants’ enzymes, polygalacturonase [54], pectinmethylesterase, and lyase, during ripening affects the texture and juiciness of the fruit [18,31]. The sensory studies reveal that potential exists for product development and commercialization of the plant. 

### 4.4. Antinutritional and Toxicological Properties of Strychnos spinosa

Antinutritional properties have an adverse effect on the food digestion in the light of the food classes, such as protein and carbohydrates, and decrease the bioavailability of minerals, such as iron and zinc [49,54,55]. The reported components of such in *Strychnos spinosa* were low and below the established toxic level [56,57]. The seeds of *Strychnos spinosa* contain strychnine and are bitter tasting [31,58]. Toxic alkaloids are present in the seeds and unripe pulp of *Strychnos spinosa* [58].

## 5. Postharvest Handling, Preservation, Storage, and Processing of *Strychnos spinosa*

### 5.1. Postharvest Handling

*Strychnos spinosa* fruits are harvested by shaking, hitting, knocking, or plucking the trees [18]. On the other hand, unripe *Strychnos spinosa* fruits are harvested and buried under a light sand for months, until it is ripe, in order to prevent postharvest losses [16,26,59]. The fruit pulp usually changes from its dry texture to a golden color after storage and, hence, is ready to be consumed [18]. As applicable with other climacteric fruits, during storage, *Strychnos spinosa* increase in soluble solid content and accumulate glucose, sucrose, and fructose [26]. The slow spoilage attributed to the fruit can be linked to the hard texture that assists in resisting insects and pathogens [16,60,61,62].

### 5.2. Products Preservation

*Strychnos spinosa* can be processed to dried products, but the preparation methods and conditions vary across locations in a small-scale level. Postharvest processing of *Strychnos spinosa* can be achieved through drying, juicing, maceration, and cooking. Although, storage influences the bioavailability and physical characteristics of the plant [63]. In southern Africa, *Strychnos spinosa* fruits are often dried by fire and or direct sunlight too, and thereafter grinded into flour [18]. Additionally, the sun-dried *Strychnos spinosa* pulp can be kept for 2 months to 5 years, making heat-drying a good preservation method for the rural communities [64]. The moisture content of *Strychnos spinosa* fruit ranges from 60 to 91%, which mainly depends on the degree and method of heating [56,65]. In addition, a properly dried fruit product does have a residual moisture content that ranges between 18 and 24%, with a good shelf-life [66,67].

### 5.3. Advantages and Challenges of Processing Techniques

**Figure 3:**
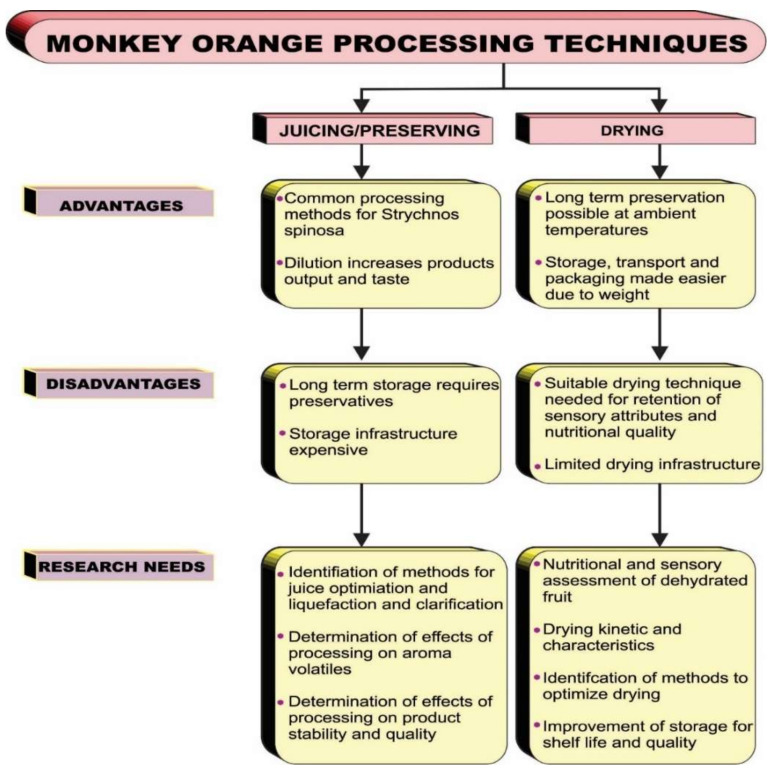
Products, processing, and way forward for *Strychnos spinosa*.

Currently, the impact of processing *Strychnos spinosa* and the assessment of its contribution to nutrient uptake is not well documented. Therefore, optimization of the processing and profiling of the food value of *Strychnos spinosa* and its products is important for the improvement of the processing procedures, which has the potential to increase the demand for the plant and its products (Figure 3). On this basis, we have identified several advantages, disadvantages, and recommendations for processing *Strychnos spinosa*. Considering the nutritional quality of the fruit, it may easily serve as an important source of nutrients for children and pregnant women [17,54,68]. Thus, improved processing of *Strychnos spinosa* fruit could be a sustainable solution to the problems of the rural communities [18].

### 5.4. Nutritional Quality and Economic Potential of Strychnos spinosa

*Strychnos spinosa* fruit and its byproducts can contribute to the economy and rural livelihood in Africa. This undervalued plant has potential that can make it withstand market competition with respect to exotic fruits (e.g., orange and strawberry). The high nutritional components and diverse phytochemicals in the plant confer immense benefits. Hence, large-scale production, marketing, and trading of *Strychnos spinosa* fruit remain important for sustainable livelihood and economic development, especially in the rural communities. Presently, there is paucity of knowledge, with limited literature on the several aspects of the fruit [69]. The commercialization of *Strychnos spinosa* will remain low until the economic returns on investment associated with the domestication of the fruit tree are profitable [70].

## 6. Domestication of *Strychnos spinosa*, Cultivation Problems, and Way Forward

**Figure 4:**
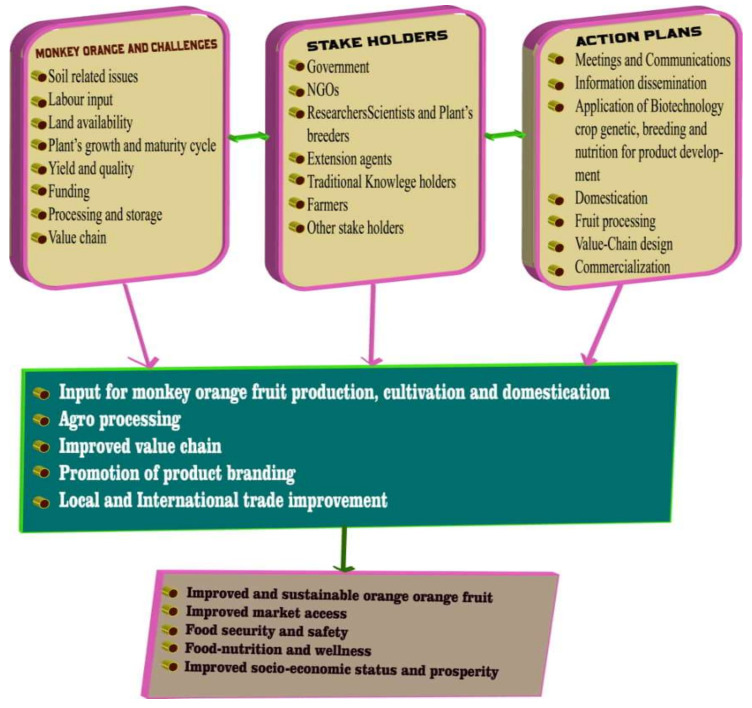
Schematic framework of priority areas for intervention on *Strychnos spinosa*.

*Strychnos spinosa* has been cultivated in southern Africa but without tangible results [9,71]. To date, no trials of the cultivation of *Strychnos spinosa* have been conducted in Africa; hence, the fruit tree is mainly sourced from the wild populations. The problems experienced by the rural populations concerning the cultivation of the underutilized fruit as a crop are: (1) land available, (2) slow growth cycle, minimal yield, and (3) common fast-cash economic culture [17,72]. Enhanced and effective information dissemination, including findings and activities, may improve as more stakeholders participate (Figure 4).

There is need for active and effective collaborations by the stakeholders on *Strychnos spinosa*. Research findings on the plants can be disseminated to the rural communities, through local NGOs and other relevant stakeholders, such as the agricultural extension services. Improving processing of *Strychnos spinosa* can enhance the possibilities for its domestication, agro-processing, production, and commercialization [29]. These envisaged findings will be useful to *Strychnos spinosa* and the much-needed intervention in research of indigenous fruit trees.

### Areas for Further Research

Sensory and nutritional composition of *Strychnos spinosa* during storage is not available. There is paucity of information on the suitability of the drying methods for *Strychnos spinosa*. Therefore, further studies on the suitability of dried products and characteristics need to be conducted to establish a drying method that fits local conditions and the possibility for commercialization. Furthermore, few studies have evaluated the nutritional and sensorial characteristics of fresh *Strychnos spinosa* juice [18]. Therefore, improving the production processes of *Strychnos spinosa* through preservation technique optimization needs to be investigated. Exploration of the value chain to enhance the economic value and potential of *Strychnos spinosa* is needed. Finally, research by the plant scientists and breeders on the domestication of *Strychnos spinosa* needs to be given more priority, owing to its commercial, nutritional, and economic potential.

## 7. Conclusions and Recommendations

*Strychnos spinosa* fruit have the potential to impart livelihood benefits and improve the nutritional status, as well as the economic prosperity, of the rural population. The micronutrients and macronutrients in the fruit tree are key to its relevance. On this basis, *Strychnos spinosa* is an important food source for children, pregnant women, and the poor. Nonetheless, limited research has been conducted regarding the value addition and processing for *Strychnos spinosa* in comparison with many popular and commercial fruits. The plant has great potential in the African rural communities, since the local environmental conditions are appropriate for its cultivation. In order to mitigate some of the existing challenges affecting the domestication of the plant for commercialization, there is need for trans-disciplinary research by different stakeholders, as well as the suggested action plan to improve the problems associated with the cultivation of the plant. Overall, we proposed priority areas for policy and intervention, and recommend an all-inclusive and sustainable development approach, as *Strychnos spinosa* could contribute to the attainment of the food-nutrition target of the United Nations Sustainable Development Goals (UN SDG, 2030).

## Figures and Tables

**Table 1:** Selection criteria applied for the selection of literature in this review.

Exclusion	Description
Underutilized African fruit plants	Existing studies on different edible and non-edible fruit plants
Underutilized southern African fruit plants	Literature on different edible indigenuos fruits of southern Africa
History and horticulture	Resarch publications on origin, taxonomy, morphology, uses, domestication, and cultivation of indigenous fruits
Chemical composition	Papers on the chemical composition and use of indigenous fruits
Non-edible uses	Literature describing uses of indigenous fruits
Inclusion	Explanation
Main subject is food nutrition and economic potential of *Strychnos spinosa* fruit tree	Nutrition literature, uses, chemicals, and prospects of *Strychnos spinosa*
Description, distribution, and ecology of *Strychnos spinosa*	Articles on distribution, taxonomy, morphology, and distribution of *Strychnos spinosa*
Diverse uses of *Strychnos spinosa*	Articles documenting the uses of *Strychnos spinosa*
Nutritional and phytochemical content of *Strychnos spinosa*	Nutritional, phytochemicals contents of *Strychnos spinosa*
Postharvest handling, preservation, storage, and processing of *Strychnos spinosa*	Articles on postharvest, preservation, and processing of *Strychnos spinosa*
Challenges, domestication of *Strychnos spinosa*, cultivation problems, and way forward	Domestication of *Strychnos spinosa*. Articles on food value chain, trade, economic prospects of plants, markets, supply chains, policy, and interventions.

**Table 2:** Proximate, vitamin C, and mineral composition of *Strychnos spinosa* fruit.

Component	Content Based on Amarteifio and Mosase [36]
Proximate and vitamin C composition	
Dry matter	19.7 (%)
Ash	4.6 (%)
Crude protein	3.3 (%)
Fat	na
Fibre	na
Acid detergent lignin	4.4 (%)
Acid detergent fibre	6.1 (%)
Neutral detergent fibre	6.2 (%)
Total carbohydrate	na
Energy value (kJ/100 g)	na
Vitamin C	88 (mg/100 g)
Total soluble sugar (%)	na
Total sugar	na
Total acidity	na
Mineral composition (mg/100 g FW)	
Phosphorus	66
Calcium	56
Magnesium	49
Iron	0.11
Potassium	1370
Sodium	21.7
Zinc	0.22
Copper	na
Manganese	na

Note: na = not available, FW = fresh weight.

**Table 3:** Overview of phytochemicals in *Strychnos spinosa*.

Plant Part	Examples of Phytochemical
Leaves	Glycosides, tannins, saponins, anthraquinones, steroids, alkaloids, and terpenoids [24,47,49]
Branches	Tannins, flavonoids, terpenoids, saponin, steroids, glycosides, and phenols [50,51]
Stem bark	Tannins, saponins, anthraquinones, steroids, alkaloids, glycosides, and terpenoids [24]
Seed	Alkaloids, tannins, phenols, phlobatannins, and steroids [52]
Fruit pericarp	Alkaloids, terpenes, sterols, fatty acids, flavonoids, and saponin [53]
Root-bark	Alkaloids, glycosides, steroids and terpenoids, tannins, anthraquinones, phlobatannins, and saponins [37,50]

**Table 4:** Sensory properties in the *Strychnos spinosa* fruit.

Properties	Description
Taste	Tarty/fermented acid-sweet [18,24]
Aroma volatiles	Major compound (>75%): trans-isoeugenol—4.762 mg/g FW [18,24] Other compounds: eugenol—307 µg/g FW; chavicol—172 µg/g FW; ρ-trans-anol—647.5 µg/g FW; 123.5 µg/g FW [31]
Aroma	Clove [41]
Texture	Not available [41]
Color	Yellow [28,31]
Acidity	0.77 [41]
pH	2.6–3.33 [31] 3.96 [18,24]
